# Small Bowel Obstruction Due to Abdominal Cocoon Syndrome in Post-COVID-19 Patients

**DOI:** 10.7759/cureus.53564

**Published:** 2024-02-04

**Authors:** Mohammad Aboelnaga, Mohamed S Elkadi, Islam E Abdelhady, Yomna H Elwan

**Affiliations:** 1 Pediatric/General Surgery, Mansoura New General Hospital, Mansoura, EGY; 2 General Surgery, Mansoura International Hospital, Mansoura, EGY

**Keywords:** covid-19, obstruction, intestinal, cocoon, abdominal

## Abstract

Background: Abdominal cocoon syndrome, also recognized as encapsulating peritoneal sclerosis, is an exceedingly rare medical condition characterized by an anomalous membranous envelopment of the small bowel. Despite its clinical rarity, the precise etiology and pathogenic mechanisms of this syndrome remain elusive.

Methods: This comprehensive discussion presents a case series encompassing six patients who sought medical attention in the Emergency Room, all sharing a common presentation: severe abdominal distension and persistent constipation. Intriguingly, these symptoms manifested following recent severe coronavirus disease 2019 (COVID-19) infections. Remarkably, none of these patients had significant medical histories or had undergone prior surgical interventions, rendering their cases even more enigmatic.

Results: The defining feature across all six cases emerged during exploratory laparotomy, where a consistent finding stunned the surgical team: the discovery of a thick, fibrous membrane enveloping segments of the small bowel. The surgical procedure entailed meticulous dissection and subsequent removal of this membrane, with tissue samples dispatched for histopathological evaluation. This diagnostic approach conclusively confirmed the presence of abdominal cocoon syndrome in each of these patients.

Discussion: The focal point of our discussion revolves around a potential connection between recent severe COVID-19 infection, intensive care unit admissions, and the subsequent development of abdominal cocoon syndrome. This intriguing association compels further inquiry to unveil the precise pathogenesis of this syndrome, particularly within the context of COVID-19. Given the diagnostic complexities associated with abdominal cocoon syndrome, this report underscores the indispensability of maintaining a heightened clinical suspicion and the significance of rigorous intraoperative assessments to ensure timely diagnosis and effective management.

Conclusion: Abdominal cocoon syndrome represents a rare and enigmatic medical condition characterized by its uncertain etiology. Our case series tantalizingly suggests a link between recent severe COVID-19 infection and the development of this syndrome. Nevertheless, comprehensive research endeavors are warranted to unravel the intricate mechanisms that underlie this intriguing association. In light of the diagnostic challenges associated with abdominal cocoon syndrome, this report underscores the pivotal role of exploratory surgery, in the absence of definitive radiological imaging, as the cornerstone for both diagnosis and therapeutic intervention. The pursuit of further investigations into the intricate relationship between COVID-19 and abdominal cocoon syndrome may ultimately yield the critical insights needed to demystify this complex medical enigma.

## Introduction

Abdominal cocoon syndrome, also known as encapsulating peritoneal sclerosis, is a rare and puzzling medical condition characterized by the anomalous encapsulation of the small bowel within an abnormal membrane. Historical records of this syndrome can be traced back to the 1860s [1], with documented cases exhibiting a broad spectrum of clinical presentations. These range from mild abdominal discomfort and distension to severe symptoms such as absolute constipation and acute abdomen [1,2], necessitating urgent surgical intervention due to complications like perforation or ischemia. Despite its long-standing recognition, the precise mechanisms underlying its development continue to elude us, leaving many questions unanswered [3,4].

Recent observations of an increase in cases of abdominal cocoon syndrome following the coronavirus disease 2019 (COVID-19) pandemic have ignited renewed interest in understanding potential contributing factors. While COVID-19 primarily targets the respiratory system, emerging evidence has shown its capacity to affect various organ systems. Notably, during the second wave of the pandemic, a significant subset of patients presented with exclusive gastrointestinal (GI) symptoms, diverging from the traditional respiratory symptoms associated with COVID-19. These GI symptoms, including nausea, anorexia, diarrhea, and abdominal pain, have raised questions about the possible role of the SARS-CoV-2 virus in the development of abdominal cocoon syndrome [5,6].

Given the intricate nature of abdominal cocoon syndrome and its potential association with COVID-19, it is imperative to launch a collaborative effort involving multiple medical centers to unlock this medical mystery. Sharing information and pooling resources across different institutions will be crucial in advancing our understanding of this rare condition, particularly in the context of the ongoing pandemic.

In terms of diagnostic modalities, radiological evaluation through computed tomography (CT) scans with double contrast administration (both oral and intravenous) has shown utility in aiding preoperative diagnosis [7,8]. This imaging approach provides valuable insights into the pathological changes associated with abdominal cocoon syndrome, potentially facilitating earlier recognition and intervention [9,10]. However, it is essential to emphasize that, in most instances, the definitive diagnosis is ascertained during surgical exploration [11-13].

The annals of medical history bear witness to the persistence of abdominal cocoon syndrome, an enigmatic condition characterized by the encapsulation of the small bowel within an abnormal membrane. Its clinical manifestations span a wide spectrum, ranging from mild discomfort to life-threatening emergencies, underscoring the urgency of early detection and intervention. While numerous hypotheses have been posited to explain its pathophysiology, none have provided a comprehensive understanding of this syndrome.

The recent upsurge in cases following the COVID-19 pandemic has added a new layer of complexity to this puzzle, warranting a collaborative, multi-center approach to unravel its mysteries. Radiological tools, such as CT scans with double contrast, offer promise in aiding diagnosis, but the definitive confirmation typically occurs during surgical exploration [11-13].

As we embark on this collective journey to demystify abdominal cocoon syndrome, we remain steadfast in our commitment to advancing medical knowledge and improving patient care. The fusion of clinical insights, research endeavors, and interdisciplinary collaboration holds the promise of shedding light on the elusive mechanisms underpinning this rare and enigmatic condition. Through collective efforts, we aspire to enhance our ability to diagnose, treat, and ultimately mitigate the impact of abdominal cocoon syndrome on the lives of affected individuals.

## Materials and methods

This study embarks on a retrospective cross-sectional analysis conducted at Mansoura International Hospital, Northern Egypt. The investigation, rooted in ethical principles, gained ethical approval from the Department of General Surgery, enabling the utilization of secondary data without the necessity for informed consent due to the absence of personal identification. Mansoura International Hospital, renowned for its multidisciplinary and tertiary medical care capabilities, stands as a beacon for patients referred from various governorates in Northern Egypt.

At the heart of this study lies a commitment to ethical considerations that were paramount in its planning and execution. The ethical approval granted by the Department of General Surgery underscores the careful ethical scrutiny applied to every facet of this investigation. The core data for our analysis was meticulously culled from the hospital's extensive records, which were diligently maintained in compliance with ethical standards. Intraoperative images, a valuable source of insights into the surgical interventions performed, played a pivotal role in the documentation and comprehension of the condition under investigation.

Furthermore, this study availed itself of the wealth of radiological data encompassing abdominal X-rays and CT scans with IV contrast. These diagnostic modalities offered a comprehensive view of the patient's medical profiles, contributing significantly to the depth of our analysis. Ethical considerations were scrupulously adhered to throughout ensuring that patient privacy and confidentiality were maintained rigorously.

Our research adopted a retrospective cross-sectional design, enabling us to glean insights from a wide spectrum of cases treated at Mansoura International Hospital. This approach allowed us to explore the research question with a breadth that would have been otherwise unattainable. The absence of personal identification information, a core ethical tenet in this study, obviated the need for individual informed consent. It is worth noting that the use of secondary data sources in medical research, when conducted in accordance with ethical guidelines, is a powerful means of advancing knowledge and improving patient care.

This retrospective cross-sectional analysis conducted at Mansoura International Hospital exemplifies the intersection of ethical considerations and medical research.

## Results

The retrospective cross-sectional study discussed in this narrative provides a unique insight into a group of patients who underwent urgent exploratory laparotomy due to acute intestinal obstruction or signs of a surgical abdomen, which was accompanied by intermittent episodes of intestinal obstruction spanning several days. What makes this study particularly intriguing is the common clinical feature that binds these patients together - the presence of an abnormal membrane encompassing either a segment or the entirety of their small intestine. Intriguingly, the root cause of this unusual complication appears to be a previous infection with COVID-19.

In the wake of the global COVID-19 pandemic, medical professionals have uncovered a myriad of manifestations and complications associated with the virus. While the predominant symptoms of COVID-19 are respiratory in nature, emerging evidence has pointed toward a broader range of systemic effects, including GI complications. This study adds another layer to our understanding of the multi-faceted impact of the virus.

The cases investigated in this study represent a small yet significant cohort of patients who experienced acute intestinal obstruction following their battle with COVID-19. These individuals exhibited symptoms such as abdominal pain, distension, vomiting, and obstipation, which led to their urgent laparotomies. However, the unusual feature in all these cases was the presence of an abnormal membrane in the small intestine. This abnormality can be seen as a form of intestinal adhesion, a condition where tissues become stuck together, causing blockages and functional issues.

The link between COVID-19 and the formation of this abnormal membrane raises questions about the pathophysiological mechanisms behind this phenomenon. It may be speculated that the virus's impact on the body's inflammatory response and vascular system plays a role. COVID-19 is known to cause widespread inflammation and endothelial dysfunction, which could potentially lead to tissue adhesion in the small intestine. It also underscores the need for further research into the virus's long-term effects and its potential to affect various organ systems.

The patients in this study were not only connected by the presence of an abnormal intestinal membrane but also by their shared history of COVID-19 infection. While the severity of COVID-19 symptoms can vary widely, GI symptoms have been noted in a significant proportion of patients. This study suggests that the virus may leave a lasting impact on the GI system, leading to complications that require surgical intervention.

Outcomes and follow-up

Patient demographics, presenting symptoms, surgical procedure, intraoperative findings, and final outcome are summarized in Table 1.

**Table 1:** Patient demographics, presenting symptoms, surgical procedure, intraoperative findings, and final outcome.

Patient	Age	Gender	Presenting Symptoms	Surgical Procedure	Intraoperative Findings	Final Outcome
1	25	Male	Abdominal Pain, Distension, Vomiting	Laparotomy & excision of abnormal membrane	Membrane enclosing terminal ileum & caecum	Good outcome, no significant complications
2	33	Male	Abdominal Pain, Distension, Vomiting	Laparotomy & excision of abnormal membrane	Membrane enclosing ileal loops	Good outcome, no significant complications
3	29	Female	Abdominal Pain, Distension, Vomiting	Laparotomy & excision of abnormal membrane	Membrane enclosing Jejunal & ileal loops	Good outcome, no significant complications
4	42	Male	Abdominal Pain, Distension, Vomiting	Laparotomy & excision of abnormal membrane	Membrane enclosing terminal ileum & caecum	Good outcome, no significant complications
5	38	Female	Abdominal Pain, Distension, Vomiting	Laparotomy & excision of abnormal membrane	Membrane enclosing Jejunal & ileal loops	Good outcome, no significant complications
6	46	Male	Abdominal Pain, Distension, Vomiting	Laparotomy, excision of abnormal membrane & appendectomy	Membrane enclosing terminal ileum, caecum, appendix & ascending colon	Good outcome, no significant complications apart from some serous wound discharge

**Figure 1:**
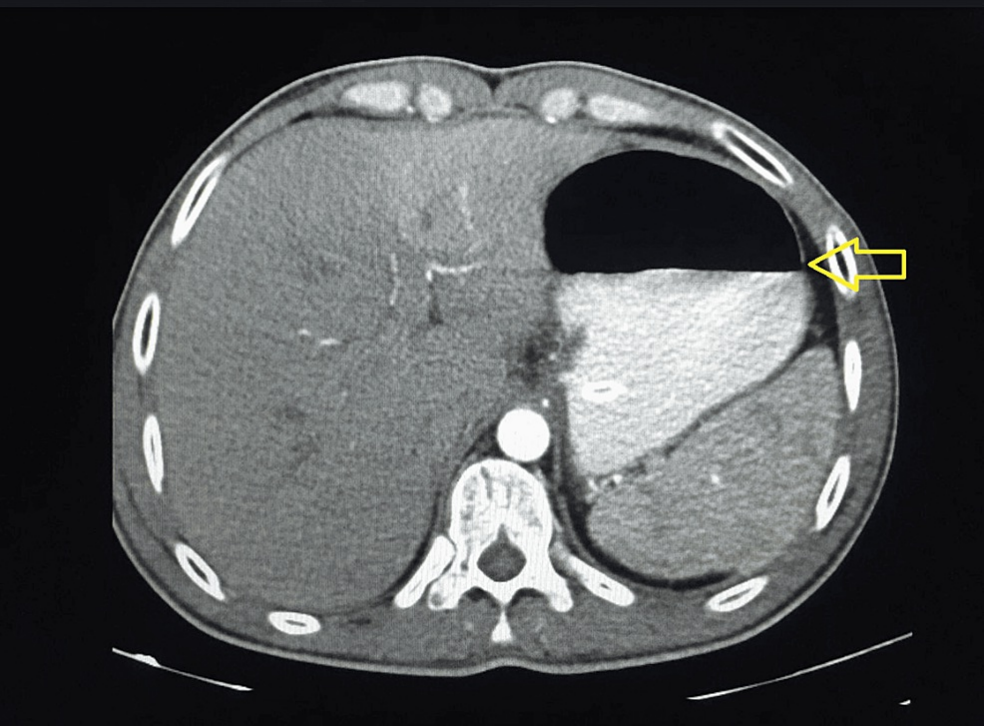
CT scan after gastrografin administration revealed a distended stomach with an air-fluid level inside.

**Figure 2:**
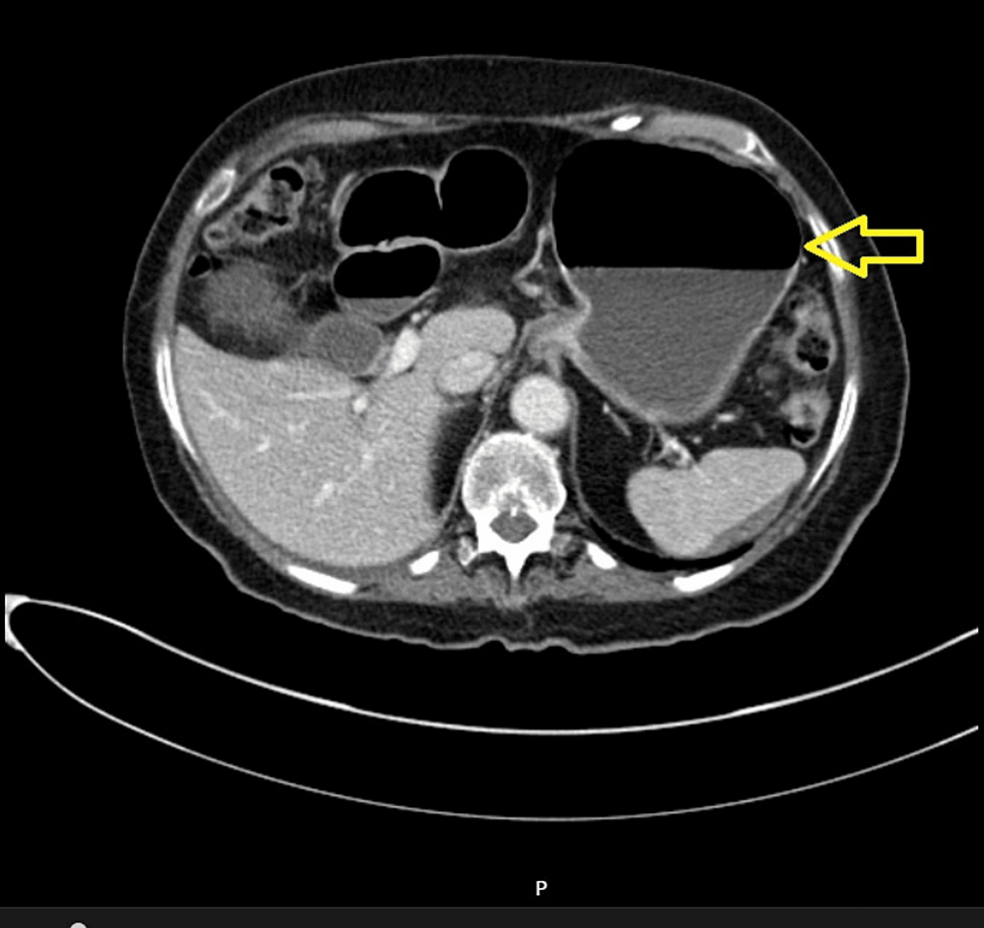
CT scan after gastrografin administration revealed a distended stomach with an air-fluid level inside.

**Figure 3:**
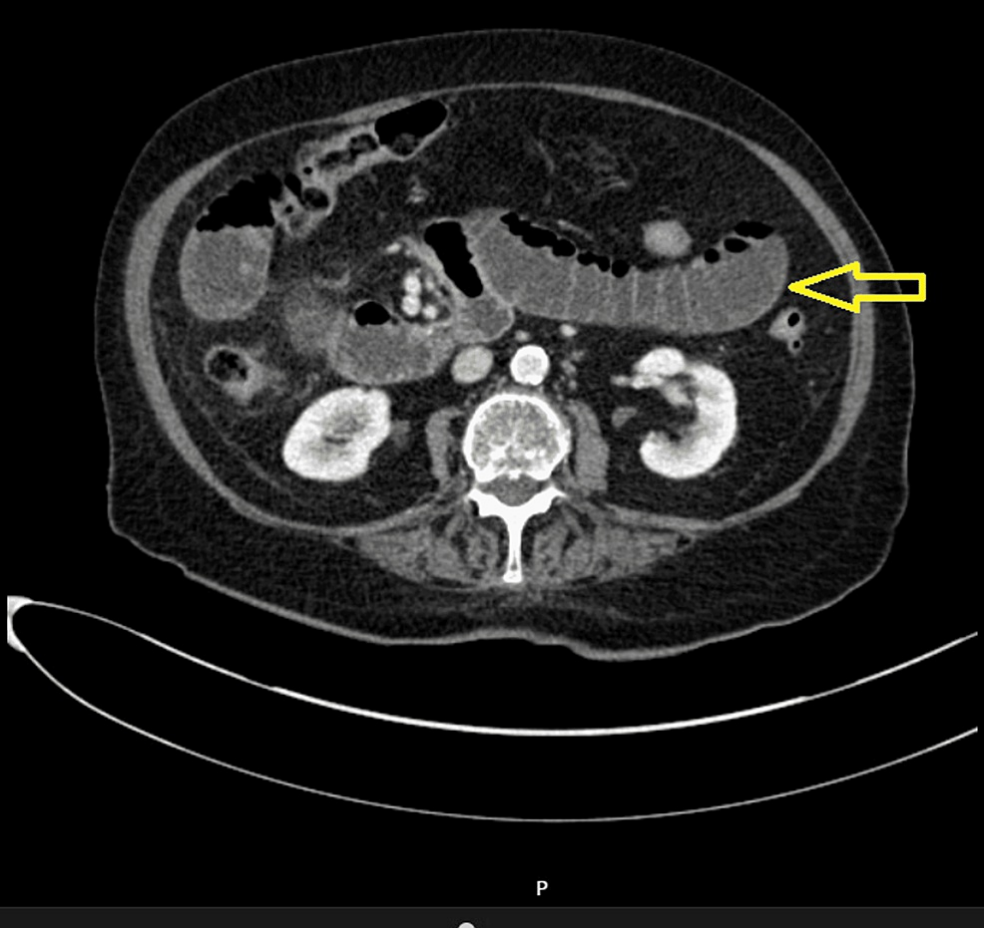
CT scan after gastrografin administration revealed distended small bowel loops with an air-fluid level inside.

**Figure 4:**
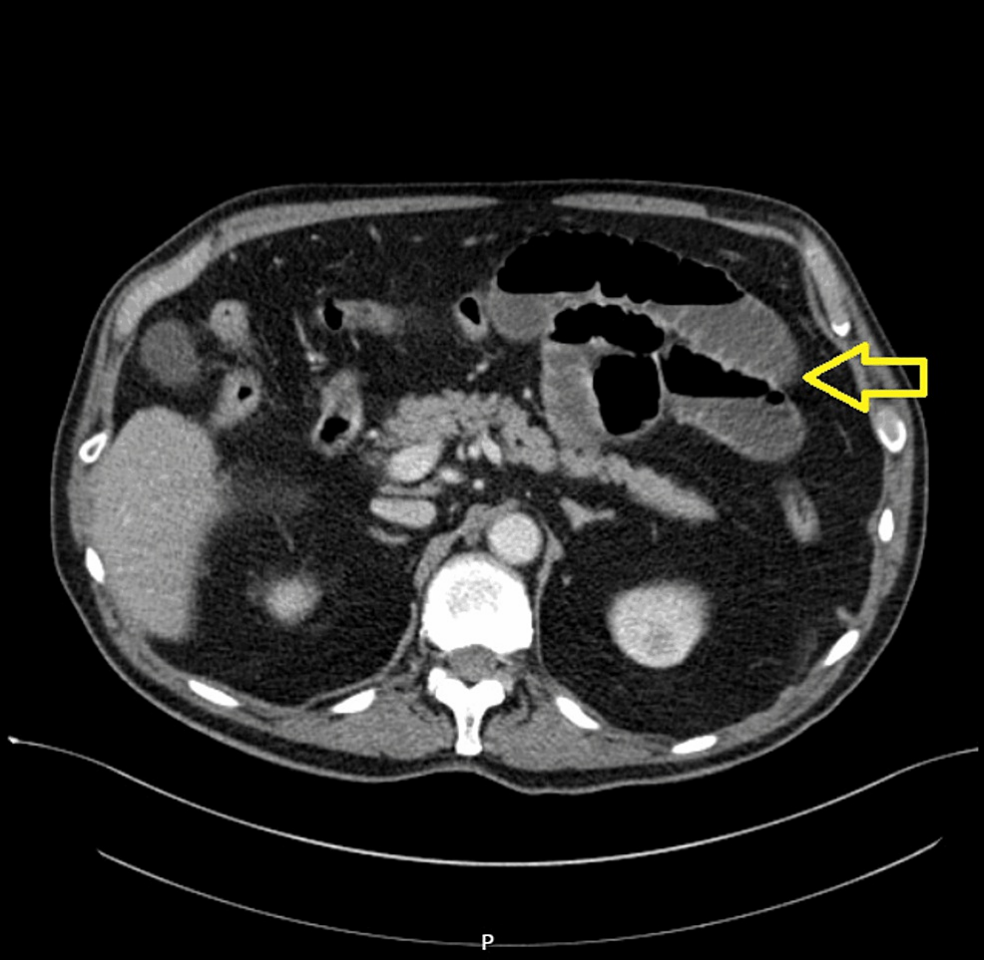
CT scan after gastrografin administration revealed distended small bowel loops with an air-fluid level inside.

**Figure 5:**
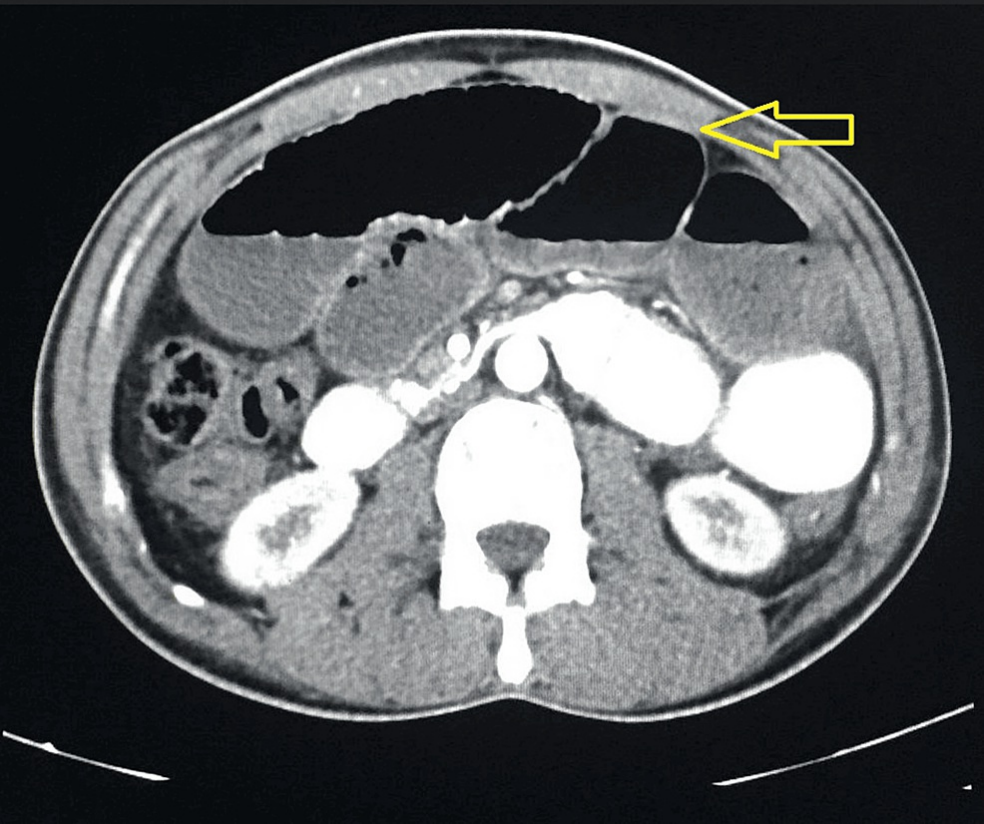
CT scan after gastrografin administration revealed distended small bowel loops with an air-fluid level inside.

Figures 1-5 show CT radiographs taken after the patient presented to the emergency department.

**Figure 6:**
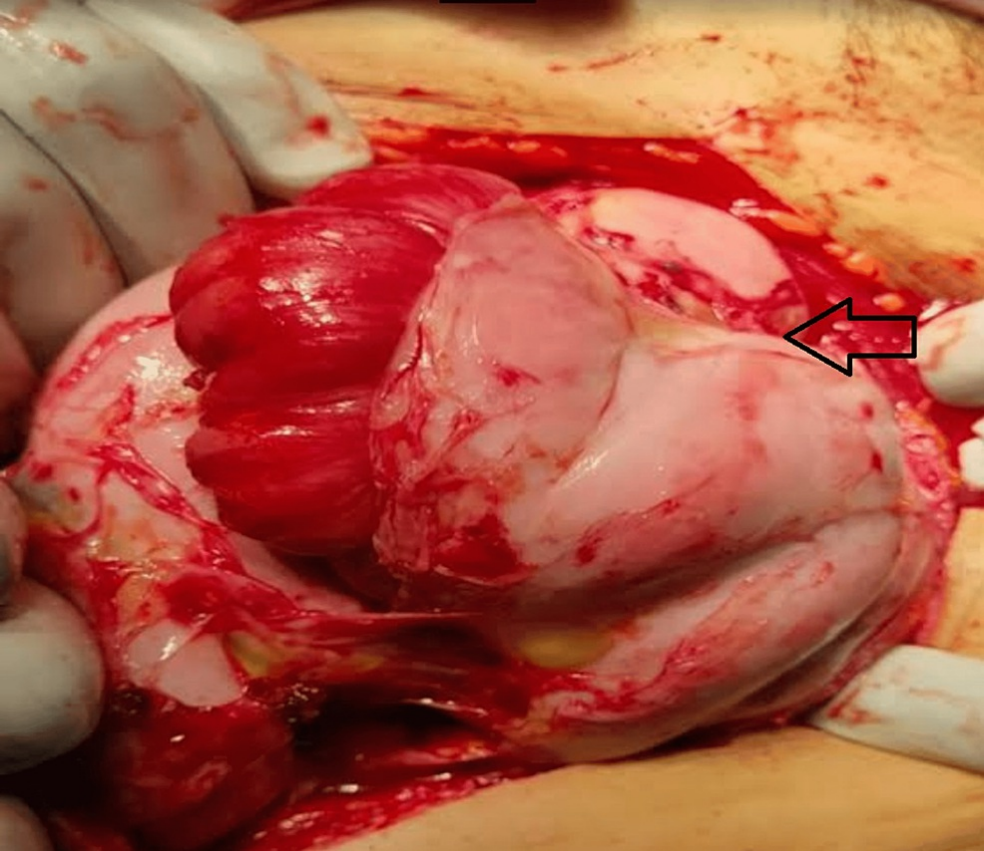
A thick membrane covering the small intestine is identified.

**Figure 7:**
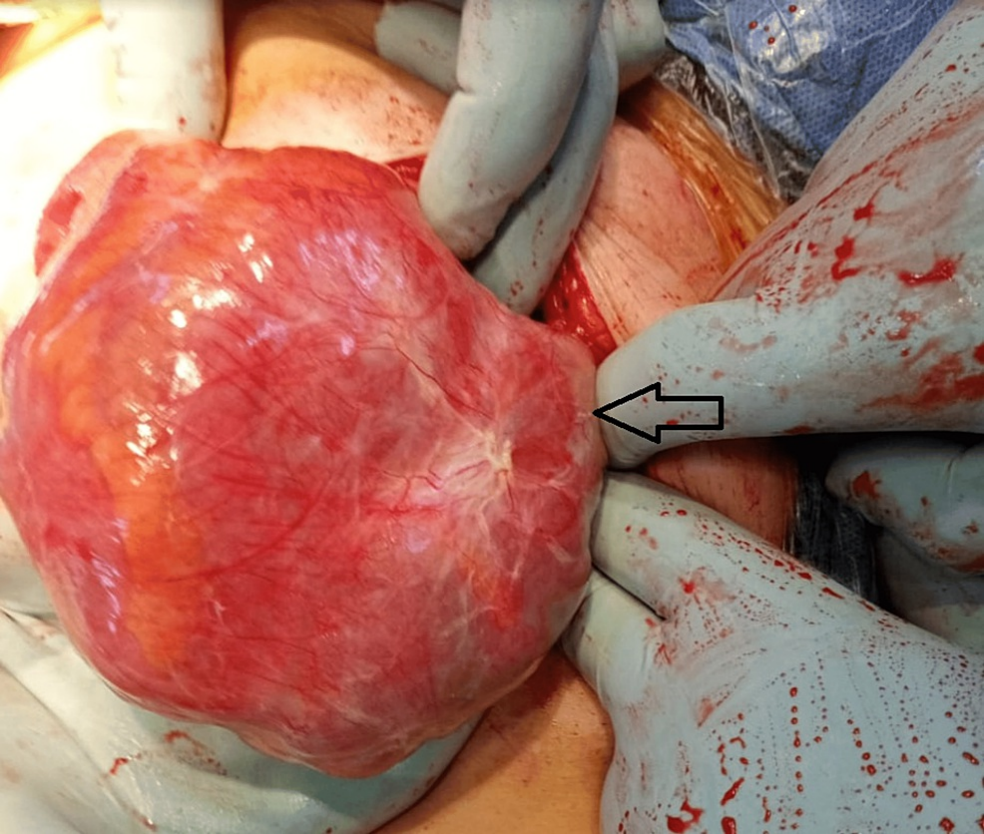
A thick membrane covering the small intestine is identified.

**Figure 8:**
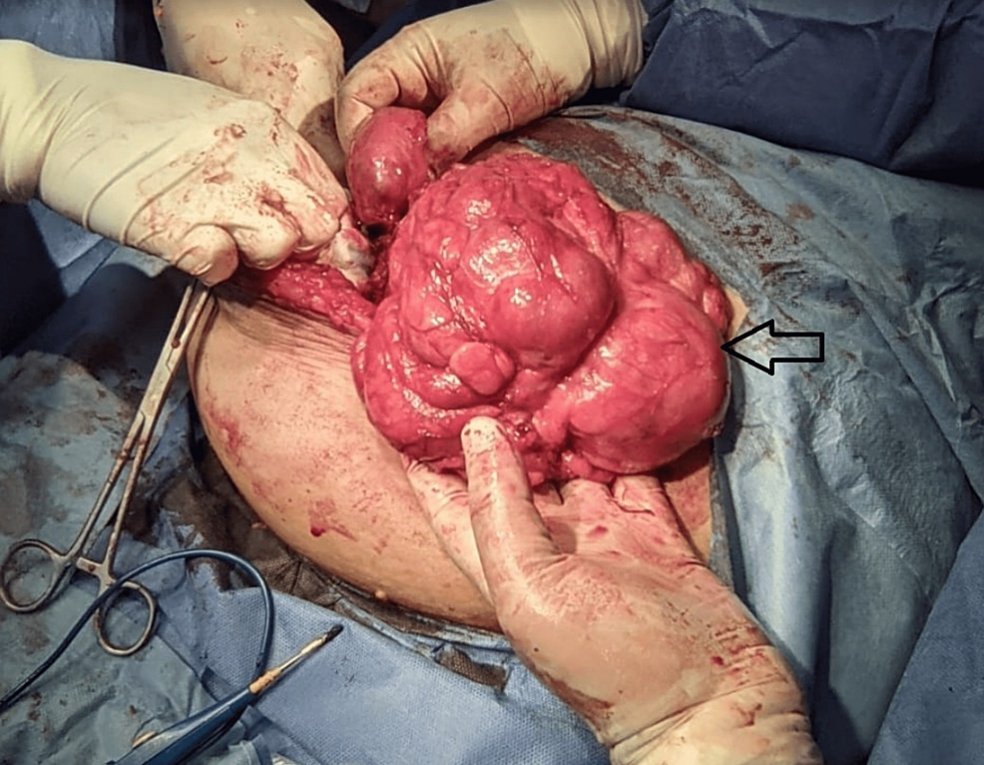
A thick membrane covering the small intestine is identified.

Figures 6-8 show intraoperative findings of the abnormal membrane covering different GIT segments.

The implications of this study extend beyond the six patients under investigation. It underscores the importance of considering COVID-19 as a multi-systemic disease with long-term consequences. GI complications are often underreported and not as widely discussed as respiratory issues, but they are just as critical. This study provides a significant contribution to the growing body of knowledge about the various ways COVID-19 can affect the body, urging clinicians to remain vigilant and consider the virus as a potential contributing factor in a wide range of health issues, including those affecting the digestive system.

## Discussion

The existence of documented cases involving an anomalous membrane enveloping the small bowel dates back to the 1860s [1]. In 1868, Cleland documented the presence of an additional peritoneal membrane, believed to originate from the yolk sac peritoneum. He coined the term "peritoneal encapsulation" and classified it as a congenital condition, as there was no discernible evidence of preceding inflammatory processes [1,2]. This membrane, characterized by its similarity in structure to the peritoneum, encases the small intestine.

An alternative hypothesis suggests that abdominal cocoon syndrome could be a variant of a well-known condition, such as Ormond disease, which primarily involves retroperitoneal fibrosclerotic alterations, leading to the entrapment of structures like the ureters [3].

The most common clinical presentation associated with this syndrome is intestinal obstruction [1,2]. Clinical signs encompass abdominal distension, abdominal discomfort or pain, and absolute constipation, as well as nausea and vomiting.

The term "abdominal cocoon syndrome" is widely used to refer to primary sclerosing encapsulating peritonitis. The term "cocoon" was introduced into the medical literature in 1978 by Foo. Despite its recognition, the precise etiology of this condition remains elusive. Notably, it exhibits a predilection for males over females [3,4]. The syndrome can be categorized into three subtypes: Type 1: Involving the encapsulation of only a segment of the small intestine; Type 2: Encompassing the encapsulation of the entire small intestine; Type 3: Extending to the encapsulation of the entire small intestine, along with portions of other organs such as the stomach, colon, liver, or ovaries.

Secondary sclerosing encapsulating peritonitis is associated with multiple risk factors, including a history of abdominal surgery, abdominal trauma, intra-abdominal inflammatory processes, and peritoneal dialysis [1,2]. These factors could potentially explain the presentation of cases following COVID-19 infection, suggesting an association between the two.

While the exact pathogenesis of abdominal cocoon syndrome remains an enigma, it is postulated that chronic irritation of the peritoneum may trigger the release of specific cytokines and stimulate macrophages to form a membrane-like structure. It is worth noting that approximately 44% of individuals experience the development of GI symptoms after fully recovering from COVID-19 [5,6]. Furthermore, the pandemic has witnessed a notable increase in the incidence of abdominal cocoon cases compared to the pre-COVID-19 era. Given that one of the most common complications of COVID-19 is pulmonary fibrosis, attributed to the virus's activation of pro-fibrotic macrophages leading to lung fibrosis, there is a plausible hypothesis that a similar mechanism might be at play in the GI tract, resulting in the formation of a fibrous membrane encasing the bowel loops. However, this remains a conjecture and necessitates rigorous histopathological studies for validation.

The establishment of a preoperative diagnosis for abdominal cocoon syndrome is a challenging endeavor [7,8]. A plain erect X-ray abdomen may reveal multiple air-fluid levels, a characteristic finding in cases of intestinal obstruction. A CT scan with double contrast has been regarded as the gold standard for preoperative evaluation of a wide range of abdominal pathologies [9,10]. However, it's essential to underscore that the definitive diagnosis is often only achieved intraoperatively during laparotomy.

Therapeutic modalities for addressing intestinal obstruction cases span a spectrum, contingent upon the severity of the condition. In milder cases, conservative measures are employed, encompassing GI rest through nil by mouth (NBM) orders, nasogastric tube placement, and total parenteral nutrition. In contrast, interventional measures come into play for recurrent and more complex cases, typically necessitating laparotomy (or laparoscopy if deemed feasible) [11,12]. The surgical approach involves the removal of the entire membrane encasing the small intestine, thereby relieving the obstruction [13].

This retrospective cross-sectional study delves into the clinical profiles of six patients who underwent exploratory laparotomy for acute intestinal obstruction or exhibited signs of a surgical abdomen. The common denominator among these cases was the presence of an abnormal membrane enveloping a segment or the entirety of their small intestine, alongside a documented history of COVID-19 infection. This enigmatic condition has historical roots dating back to the 1860s, with hypotheses ranging from congenital origins to potential associations with other known diseases. Its most prevalent manifestation is intestinal obstruction, accompanied by a spectrum of clinical signs.

Abdominal cocoon syndrome is often used to describe primary sclerosing encapsulating peritonitis. Its underlying cause remains elusive, with a notable male predilection. The syndrome can be categorized into three distinct types based on the extent of small intestine encapsulation. Secondary sclerosing encapsulating peritonitis is associated with various risk factors, some of which may provide insight into the emergence of cases post-COVID-19 infection.

The pathogenesis remains incompletely understood, yet a recent study revealed a higher prevalence of anterior pituitary deficiencies in individuals experiencing long COVID [14]. The hypothesis involving endocrine issues, specifically anterior pituitary insufficiency, may offer an explanatory framework for understanding long COVID ultimately contributing to persistent peritoneal irritation that might be conjectured to influence membrane formation. The confluence of increased GI symptoms post-COVID-19 recovery [15], a rise in cocoon cases during the pandemic, and the well-documented pulmonary fibrosis associated with COVID-19 raises intriguing hypotheses regarding potential GI fibrosis mechanisms.

Diagnosis of abdominal cocoon syndrome is challenging, often necessitating intraoperative confirmation. Radiological evaluation through X-rays and contrast-enhanced CT scans aids in preoperative assessment.

Therapeutic interventions for intestinal obstruction range from conservative measures in milder cases to surgical interventions, primarily laparotomy or laparoscopy if feasible, for more complex scenarios. Surgical removal of the encapsulating membrane constitutes the cornerstone of treatment.

This study sheds light on the clinical presentation, historical context, potential associations, and diagnostic challenges surrounding abdominal cocoon syndrome. The enigma persists, motivating further research endeavors to unravel its intricacies and enhance patient care.

Limitations of the study

The limited sample size, comprising only six cases, poses potential challenges to the generalizability of the findings. Access to radiographic scans and intraoperative images may not always be readily available, which is a notable constraint in this study. This research heavily relies on medical records and retrospective data to establish connections between operative findings, presenting symptoms, and medical history, and the quality and accessibility of these records present inherent limitations. Establishing causation can be a formidable task. While this study may reveal correlations, definitively determining causality is often elusive. Hence, collaborative efforts involving multiple medical centers are imperative to substantiate the outcomes of this study.

## Conclusions

While intestinal obstruction attributed to abdominal cocoon syndrome remains exceptionally rare, it warrants consideration as a potential etiology in cases of chronic abdominal pain and bowel obstruction, particularly when more commonplace causes have been ruled out. Recent COVID-19 infection may serve as a noteworthy indicator, albeit necessitating heightened collaboration in data collection and information exchange among various medical centers. For preoperative diagnosis, the foremost imaging modality is the CT scan with double contrast, offering valuable insights into the condition. However, it is essential to acknowledge that the definitive diagnosis is frequently achieved intraoperatively during exploratory laparotomy.

In summary, while abdominal cocoon syndrome leading to intestinal obstruction is an infrequent occurrence, it should not be dismissed when evaluating patients with chronic abdominal pain and bowel obstruction. Recent COVID-19 infection may raise suspicion, but comprehensive data sharing among healthcare institutions is imperative for a more thorough understanding of this potential association. The CT scan with double contrast is a valuable tool for preoperative assessment, yet the ultimate diagnosis is often made during surgical exploration. Continued research and clinical collaboration are essential to enhance our comprehension of this rare condition and improve patient care.
